# Current and Future Applications of 5-Aminolevulinic Acid in Neurosurgical Oncology

**DOI:** 10.3390/cancers17081332

**Published:** 2025-04-15

**Authors:** Jia-Shu Chen, Jacob S. Young, Mitchel S. Berger

**Affiliations:** Department of Neurological Surgery, University of California San Francisco, San Francisco, CA 94143, USA; jia-shujoseph.chen@ucsf.edu (J.-S.C.); jacob.young@ucsf.edu (J.S.Y.)

**Keywords:** 5-aminolevulinic acid, extent of resection, glioma, meningioma, metastasis, survival

## Abstract

Maximal safe surgical resection is the gold standard in brain tumor surgery. Fluorescence-guided surgery (FGS) is one of many intraoperative techniques that have been designed with the intention of accomplishing this goal. 5-aminolevulinic acid (5-ALA) is one of the main fluorophores that facilitates FGS in neurosurgical oncology. Multiple different types of brain tumors can take in and metabolize 5-ALA into protoporphyrin IX (PpIX) through the mitochondria heme biosynthesis pathway. PpIX then selectively accumulates in brain tumor cells due to decreased ferrochelatase activity and emits red fluorescence (630–720 nm) when excited with blue light (375–440 nm). This mechanism allows neurosurgeons to better visualize tumor burden and increase extent of resection while preserving non-cancerous brain parenchyma and, specifically, eloquent white matter tracts, if combined with mapping techniques, thereby minimizing morbidity while improving survival. While 5-ALA use is well established in the treatment of high-grade gliomas, its applicability in recurrent high-grade and non-enhancing IDH-mutant low-grade gliomas, as well as non-glial tumors, is less established or limited by certain features of their cellular and molecular biology. This review aims to discuss the current landscape of 5-ALA utility across the diverse range of brain tumors, practical considerations that optimize its current use in neurosurgery, modern clinical limitations of 5-ALA, and how its application can be expanded by combining its use with other techniques that overcome current limitations.

## Simple Summary

Maximal safe surgical resection is the cornerstone of neurosurgical oncology. Fluorescence-guided surgery with 5-aminolevulinic acid (5-ALA) is an intraoperative neurosurgical technique that enhances visualization of tumor burden in real-time, which helps increase extent of resection. Several key clinical trials and institutional experiences have advanced the use of 5-ALA for high-grade gliomas, and there has been a recent increase in its utility for low-grade gliomas and meningiomas. This review examines the existing literature on 5-ALA in the treatment of brain tumors and summarizes strategies that optimize its efficacy as well as its limitations and areas of future research that can further improve its utility in neurosurgical oncology.

## 1. Introduction

Maximal safe surgical resection is the foundational principle in brain tumor surgery, with improved overall and progression-free survival associated with greater extent of resection across all brain tumor types [1,2,3,4,5]. One of the greatest barriers to gross total resection in high-grade gliomas (HGGs) and glioblastoma, the most prevalent types of primary malignant brain tumors, is their infiltrative nature [6]. Microscopic invasion of tumor cells into the surrounding brain parenchyma creates diffuse tumor borders that are not visible radiographically or to the naked eye [7,8,9,10,11]. It is well known that glioblastoma and HGGs have significant intratumoral heterogeneity, with tumor cells at the periphery possessing specific cellular mechanisms that facilitate neuronal hijacking and microtubule formation, which enable tumor cell migration and chemo-/radio-resistance [12,13,14]. Leaving these tumor cells behind unintentionally at the time of surgery thereby ensures tumor recurrence and faster time to mortality [15]. Understanding these biological mechanisms and their clinical implications has led to the concept of supratotal resection, where the resection is intentionally extended beyond the visible and contrast-enhancing tumor regions to eliminate microscopic disease. While there is convincing evidence that supratotal resection increases progression-free (PFS) and overall survival (OS), it is not without its risks as there is a greater likelihood of iatrogenic neurological injury from resection of healthy cortical and subcortical tissue when not assisted by adjunctive techniques that discriminate between functional and non-functional tissue [16,17,18,19,20,21,22]. Iatrogenic neurologic deficits are also associated with worse outcomes, which means that it is essential to have a guided approach to achieving a resection that includes microscopic disease instead of a blind supratotal resection [23,24]. Many intraoperative modalities have been developed to facilitate the former, including neuronavigation, functional brain mapping, intraoperative MRI, and ultrasound. However, fluorescence-guided surgery (FGS) with 5-aminolevulinic acid (5-ALA) has become one of the most reliable modalities. Several investigations have compared the modalities, most notably, a recent randomized trial that evaluated 5-ALA versus intraoperative MRI and demonstrated comparable rates of complete resection and postoperative survival but a shorter operative duration in the 5-ALA cohort [25]. This highlights the advantages of 5-ALA that attract its use, which include low cost, high logistical feasibility, a good safety profile, and real-time intraoperative guidance that accounts for brain shift from resection.

The concept of FGS is based upon systemic administration of a fluorescent agent (fluorophore) that leads to its broad distribution but selective uptake or metabolization by neoplastic tissue. The agent and its metabolites then accumulate and fluoresce only in cancer cells under exposure to specific light wavelengths, allowing for a visible distinction from healthy tissue. The selective fluorescence provides a visual cue that helps surgeons decide whether tissue should be resected or not. Several fluorophores have been used in neurosurgery, including 5-aminolevulinic acid (5-ALA), fluorescein, and indocyanine green (ICG). While fluorescein and ICG have applications dating farther back than 5-ALA, 5-ALA has gained the most traction in the treatment of brain tumors, especially glioblastoma and HGGs, because of its clinical efficacy. 5-ALA is a metabolic precursor to protoporphyrin IX (PpIX) in the hemoglobin biosynthesis pathway. In non-cancerous cells, PpIX is converted into heme through ferrochelatase (FECH) activity. However, in tumor cells like HGG, FECH activity and intracellular concentration of ferrous iron (Fe^2+^) are significantly reduced, which leads to the intracellular accumulation of PpIX [26,27,28]. The selective buildup of PpIX is further perpetuated by upregulation of aminolevulinic acid-dehydratase and porphobilinogen-deaminase, which facilitate the conversion of 5-ALA to PpIX, as well as downregulation of the ABCG2 transporter, which coordinates extracellular efflux of PpIX [29,30,31]. Once peak distribution and intracellular accumulation are reached, tumor cells can selectively emit red fluorescence (~635 nm) under excitation of blue light (375–440 nm) [32]. In 1998, Stummer et al. demonstrated that HGG cells exhibit selective 5-ALA fluorescence, similar to in gastrointestinal, bladder, and skin cancers, which were shown earlier [33]. Following that report, in 2006, Stummer et al. published the results of a landmark trial comparing conventional microsurgery with white light to FGS with 5-ALA in HGGs, which found that the 5-ALA cohort had significantly greater extent of resection and 6-month PFS without higher rates of adverse events [34]. In the two decades since the trial, there has been a significant increase in the clinical utilization and research of 5-ALA for brain tumors to better understand how to maximize its efficacy. While several fluorophores are utilized in neurosurgical oncology, this review focuses on the modern applications and protocols for 5-ALA since it is the most prominently used agent, as well as its clinical limitations, to help guide the design of future research studies that may help optimize its implementation in brain tumor surgery. Additionally, 5-ALA is generally the fluorophore of choice given its superior sensitivity and specificity and FDA approval for HGGs [35,36]. A description of the most commonly used fluorophores in neurosurgery and their different applications and advantages can be found in Table 1.

## 2. Current Neurosurgical Applications of 5-ALA

### 2.1. Primary and Recurrent High-Grade Glioma

The most common use of 5-ALA in brain tumor surgery is for the resection of HGGs, as evidenced by its recent FDA approval in 2017. Since the landmark 2006 trial, numerous studies have replicated its efficacy, with 5-ALA increasing median OS by 3–6 months and rates of gross total resection by 25–30% compared to conventional microsurgery [37,38,39,40]. Much of the efficacy of 5-ALA in HGG is attributed to the high sensitivity, specificity, and diagnostic accuracy of 5-ALA fluorescence in HGG. Across multiple studies correlating intraoperative fluorescence to histopathology, 5-ALA had a sensitivity between 81 and 92%, a specificity between 80 and 94%, and an accuracy between 92 and 96% [41,42]. The high fidelity between fluorescence and cancerous tissue allows neurosurgeons to have increased diagnostic confidence in the resection boundaries, which allows for greater extent of resection without compromising non-cancerous brain tissue. This helps explain how the majority of patients treated with 5-ALA had fewer neurological deficits (42.2%) or the same incidence of neurological deficits (34.5%) when compared to conventional microsurgery in several studies [39,43].

These advantages are not exclusive to primary HGG but also extend into the setting of recurrent HGG. Under conventional settings, re-resection of recurrent HGG is especially challenging as scar formation, gliosis, and peritumoral reactive changes to adjuvant treatment including chemotherapy, radiotherapy, and targeted treatments obscure visual interpretation of tumor and non-tumoral tissue intraoperatively [44]. In 2009, Arya et al. demonstrated for the first time that 5-ALA fluorescence persists in recurrent tumor tissue with good reliability, as the positive predictive value for fluorescence in both pathologically appearing (99.5%) and normal-appearing (93%) tissue regardless of fluorescence-strength was very high [45]. This was an important finding as it proved that the molecular biology driving protoporphyrin synthesis, accumulation, and fluorescence is preserved during the transition from primary to recurrent tissue and even under the physiologic stress of surgical resection, chemotherapy, and radiation treatment. This proof-of-concept study pioneered the way for further investigations into the utility of 5-ALA in recurrent HGG, which have redemonstrated improved survival and extent of resection [46,47,48].

On the topic of 5-ALA in HGG, it is important to note that 5-ALA primarily serves as a diagnostic adjunct to help neurosurgeons make informed intraoperative decisions and not a primary treatment modality. The use of 5-ALA for photodynamic therapy (PDT) has been proposed, and research is ongoing to determine how 5-ALA can serve as a primary treatment through this mechanism. Photodynamic therapy is a treatment where photosensitizing compounds accumulate intracellularly and are photoactivated via a specified wavelength of light, which leads to the catalyzation of cytotoxic reactive oxygen species that induce local tissue destruction. Several clinical trials have designed different protocols for 5-ALA PDT in conjunction with FGS compared to conventional surgery, and in isolation for non-operable HGGs [49,50]. Additionally, in the past year, 5-ALA has also been engineered into a sonodynamic therapy (SDT) where its intravenous administration in conjunction with focused ultrasound stimulates cytotoxic reactive oxygen species intratumorally for tumor cell killing [51]. While these studies have demonstrated good safety and promising efficacy, more research must be performed as these trials are underpowered and heterogenous in study design, patient population, and protocol, and it is difficult to distinguish whether the treatment effect is due to fluorescence-guided resection or PDT/SDT.

### 2.2. Low-Grade Glioma

The strong utility of 5-ALA in HGGs raises the question of whether it also possesses the same efficacy in low-grade gliomas (LGGs). Unfortunately, the molecular composition and metabolic biology of LGGs preclude the similar use of 5-ALA. The intensity of 5-ALA fluorescence in gliomas has been shown to correlate with the tumor’s degree of contrast enhancement and proliferative index [52,53,54]. These are lower in LGGS given their decreased cellularity, lower vascularity, and a less permeable blood–brain barrier [55,56,57]. As a result, it has been reported that only 10–20% of LGGs will fluoresce with 5-ALA [57,58,59], and the fluorescence does not reliably span the entirety of the tumor but primarily anaplastic foci, if present [60,61,62]. In multiple studies, almost all WHO grade II tumors (91–100%) had no visible fluorescence, and the majority of WHO grade III tumors (80–90%) only had focal fluorescence [53,60,61,62]. Given this, 5-ALA should not be utilized as a vehicle for achieving gross total resection in LGGs as there will undoubtedly be regions of tumor that do not fluoresce and will be retained if relying solely on 5-ALA [63]. However, this does not mean 5-ALA does not still have an important role in LGG. 5-ALA possesses value in identifying tumor regions that have undergone malignant transformation and ensures that these high-risk areas are resected. Additionally, since degree of fluorescence correlates strongly with tumor histopathology, 5-ALA can be used in tumors where the grading is radiographically ambiguous so that the neurosurgeon can have a sense intraoperatively of how aggressive the tumor is. In our own institutional experience of radiographically suspected LGGs, over two-thirds were actually determined to be high-grade tumors, of which 79% expressed strong visible fluorescence [62]. This suggests that radiographic LGGs are oftentimes actually high grade in nature, and the strong concordance of 5-ALA fluorescence can help elucidate this more rapidly. By using 5-ALA as an adjunct to identify malignant regions in LGGs intraoperatively, the argument can be made that 5-ALA should help prolong PFS by ensuring that the tumor tissue most likely to cause recurrence is removed. However, there are few studies that investigate the impact of 5-ALA on survival in LGG, in large part because of its limited use in this setting, so greater research is needed to better define 5-ALA’s impact and role for this pathology.

### 2.3. Meningioma

The role of 5-ALA in the resection of meningiomas is less well defined than it is for gliomas, which has been the primary focus for the past two decades. However, interest in its application towards meningiomas has rapidly increased as experience has grown, with promising results. The first published experience of 5-ALA in meningiomas was reported by Kajimoto et al. in 2007, whose use of 5-ALA in 24 cases highlighted fluorescence in 83% of them with a specificity of 100% [64]. Most importantly, the use of 5-ALA in this series led to the discovery of residual tumor along veins and sinuses, hypertrophic dura, and skull flaps and edges in several different cases that would have otherwise gone unnoticed, thereby allowing for true Simpson grade I resection. Furthermore, maximizing the extent of resection through 5-ALA did not come at the cost of indiscriminate resection, which increases the risk of neurological and vascular injury. Since the initial series, a greater number of institutional experiences with larger sample sizes have been reported with several important and consistent findings [65,66,67]. First, meningiomas reliably fluoresce regardless of grade and with high sensitivity ranging from 90 to 100% [56]. Second, when stratifying the sensitivity of fluorescence according to peritumoral tissue site, the reliability of fluorescence in affected dura and the dural tail decreases relative to other adjacent sites such as affected bone, arachnoid, parenchyma, and vasculature. More than 50% of sampled pathologic dura throughout different studies did not have visible fluorescence with 5-ALA use [66,67,68].

The indiscriminate fluorescence regardless of histopathology in meningiomas has been attributed to high protein expression of PpIX metabolism genes including ABCB6, ABCG2, CPOX, and FECH throughout all tumor grades, which enables high-throughput metabolism and accumulation [69]. This is an important distinction as it allows for broad patient selection and generalized interpretation when using 5-ALA in meningiomas rather than the careful calculus that is required for gliomas, where the pretest probability of HGG versus LGG dictates how the fluorescence is interpreted intraoperatively. Variable dural fluorescence in meningiomas is an important finding and consideration for neurosurgeons because it informs them to remain vigilant when localizing and excising the dural tail in the setting of 5-ALA. The surgeon should not rely on 5-ALA in isolation for identifying residual dural tail and instead leverage other adjunctive intraoperative tools that enhance tissue visibility/diagnosis or quantify fluorescence beyond what is visible to the naked eye [70,71,72]. Overall, the use of 5-ALA in meningiomas is promising for its ability to guide the removal of additional tumor that was previously undetected by the naked eye, which has been reproduced across different institutional experiences. This would primarily be in the setting of tumors that are not well circumscribed and in the skull base where small areas of invasion must be differentiated from healthy neurovascular structures through small corridors. However, additional work remains before it can become a staple in the armamentarium for meningiomas. To date, there is no standardized 5-ALA protocol specifically for meningiomas, and, given that the rate of PpIX clearance in meningiomas differs from glioma, a protocol specifically tailored to meningioma kinetics must be established [73]. Additionally, the benefit of 5-ALA for OS and PFS, as well as postoperative morbidity, is unclear. Thus, the question of whether the risks of completely resecting residual tumor around vital neurovascular structures facilitated by 5-ALA outweigh the benefit of postoperative radiotherapy remains.

## 3. Patient Considerations for 5-ALA

### 3.1. Preoperative

To maximize the utility of 5-ALA and ensure safe administration for the patient, there are several important technical considerations that must be accounted for throughout the perioperative window. Preoperatively, the most important considerations are dosage, timing, and baseline health and medication regimen, all of which can alter the fluorescence intensity, clearance, and adverse events of 5-ALA. To date, the conventional dosing of 5-ALA in FGS has been oral ingestion of 20 mg/kg body weight in 50 mL drinking water, regardless of tumor type and grade. This dosing schema was designed for HGGs and has proven sufficient for evaluating 5-ALA in LGGs and meningiomas preliminarily, but it may be time to start considering tumor-specific dosing as different tumors have variable responses to 5-ALA. For HGG, there is evidence to suggest that the fluorescent intensity peaks at 20 mg/kg as the bottleneck is no longer penetrance through the blood–brain barrier but intracellular capacity for PpIX to accumulate [74]. However, in the case of LGGs where fluorescent intensity is lower and more variable, Suero Molina et al. demonstrated that doubling the dose to 40 mg/kg body weight significantly increased the fluorescence intensity and doubled the rate of cases where the visibility threshold of fluorescence (1 ug/mL) was reached [75]. This effect is facilitated by the fact that there is still reasonable integrity of the blood–brain barrier to prevent the maximal transit of 5-ALA from the bloodstream to the tumor in LGGs. For meningiomas, there is a lack of clinical trial data that test different doses of 5-ALA to guide a standardized protocol currently. Controlled comparisons of the quantified fluorescence across different 5-ALA doses in meningioma patients would provide data that can help establish standardized dosing protocols. However, in vitro and human kinetics data suggest that the 20 mg/kg dose should be sufficient to reach visible fluorescence since meningiomas express greater intensity than HGGs at the same dose [73,76]. The differences between meningiomas and HGGs are the time to visible fluorescence and total duration of fluorescence, which are shorter and longer in meningiomas, respectively. These observations raise the question of the most optimal time for oral ingestion.

In HGGs, the standard protocol is for oral administration of 5-ALA to occur 3–4 h prior to the induction of anesthesia given that the time to peak fluorescence is 7–8 h, meaning peak fluorescence will be reached by the time the neurosurgeon has resected the central tumor core and is starting to evaluate the tumor periphery and demarcating the margins. This is generally well agreed upon as all clinical trials to date have adopted this time frame for HGGs. However, there is emerging evidence that there are regional differences in time to peak fluorescence, and tumor areas of intrinsically weaker fluorescence, such as the peripheries, have a longer time to peak fluorescence ranging from 8 to 9 h [77]. Given these data, the argument can be made that oral ingestion should be 4–5 h prior to induction in HGGs since it is most critical for the surgeon that peripheral fluorescence is maximized instead of the central bulk, which is more readily recognizable in the absence of fluorescence. For LGGs, data suggest that the kinetics of 5-ALA metabolism mirror that of HGGs and timing of administration can follow the same protocol. However, the differences in meningioma kinetics warrant discussion of whether 5-ALA administration should be closer to the time of anesthesia induction. Unlike gliomas, the time to the critical portion of the tumor resection is not as long since resection usually begins following dural incision. Furthermore, if visible fluorescence is reached faster and retained for a longer time, there would be a stronger concordance between time with open dura and visible fluorescence if the 5-ALA ingestion latency period was shorter. Ultimately, more research must be performed to optimize the timing of 5-ALA administration, and one of the most important considerations should be the institution effect, where timing is tailored to the average time it takes to reach and begin debulking the tumor for each surgeon and center.

While oral ingestion of 5-ALA is benign overall, there are side effects associated with its use that both the neurosurgeon and anesthesiologist should be aware of and take into consideration when planning and counseling patients preoperatively. First, the mechanism of action that 5-ALA functions through results in porphyria-related side effects given that PpIX is a porphyrin. As a result, the most common side effects to be aware of are photosensitivity and liver injury, as well as nausea, vomiting, and diarrhea. Contraindications to 5-ALA use should include history of acute intermittent or chronic porphyria. While the elevation in liver enzymes after 5-ALA use is clinically insignificant, with no reported cases of liver failure, baseline liver function tests should be drawn to trend as elevated levels have persisted for up to 6 weeks in some cases [78]. Additionally, preoperative clearance should be taken in patients with liver dysfunction, cirrhosis, adiposity, or hepatotoxic medication use [79]. Other medications to be vigilant about include anti-hypertensive and phototoxic drugs. 5-ALA is associated with hypotension most likely secondary to elevated nitric oxide levels, which is why it is reasonable to consider holding anti-hypertensive medications 24 h preoperatively. More importantly, 5-ALA is associated with skin erythema, blistering, and peeling secondary to ambient light exposure. As a result, phototoxic medications including tetracyclines, sulfonylureas, thiazide diuretics, and other common offenders are held 24 h prior to and after surgery. Physical precautions are also taken intraoperatively and postoperatively to limit the patient’s exposure to ambient light for no longer than 48 h and decrease the risk of phototoxic events.

### 3.2. Intraoperative

In addition to limiting patient light exposure intraoperatively, there are several other important intraoperative considerations related to the optimization of fluorescent signal interpretability and accuracy. At baseline, the illumination of PpIX fluorescence through surgical microscopes is specifically titrated to a degree of maximal sensitivity to avoid false-positive signal and resection of healthy tissue. Ambient light can decrease visibility of fluorescence and sensitivity by creating background noise that washes out fluorescent signal. As a result, physically decreasing the ambient lighting has a multifactorial purpose in 5-ALA FGS. The tradeoff of reducing ambient light is the difficulty of operating under dim blue-light conditions, which requires frequent switching from blue-light excitation to standard white-light conditions. Some groups have mitigated this challenge by adding a secondary illuminant that provides light ranging from 475 to 600 nm, which does not interfere with the excitation or fluorescent wavelength or sacrifice the tumor-to-background color contrast [80]. There are also now filter mechanisms built into the microscopes that account for and block out ambient light [81], as well as headlamps that generate a more powerful and focused excitation light, alternate between white and excitation light via pedal activation, and follow the gaze of the surgeon, allowing for better visualization and a more ergonomic workflow than the microscope [82]. Another source of interference with the fluorescent signal is blood products within and around the resection cavity. Erythrocytes are capable of absorbing the blue excitation light, which decreases or completely abrogates the red PpIX fluorescence in tumor tissue [31]. Thus, it is important for the surgeon to preserve a clean surgical working environment and irrigate surrounding blood prior to evaluating fluorescence. Finally, one of the primary intraoperative challenges of 5-ALA is the detection of signal in large, deep-seated tumors where the microscope is unable to illuminate the depths of the cavity. In these situations, there are adapted instruments that increase light penetration and fluorescence emission. Morshed et al. demonstrated effective use of wavelength-specific fiber-optic lighted suction to provide excitation light into the cavity while resection is ongoing, while other groups have shown that endoscopic-assisted visualization of the cavity also enables better visualization of fluorescence deep in resection cavities [83,84,85]. Overall, with increased experience in utilizing 5-ALA over time, a better understanding of how to optimize its use has developed, as well as elucidation of its limitations and what future work must be carried out to enhance its utility. A summary of preoperative, intraoperative, and postoperative considerations when using 5-ALA in HGGs, LGGs, and meningiomas is detailed in Table 2.

## 4. Limitations and Future Neurosurgical Applications of 5-ALA

**Figure 1:**
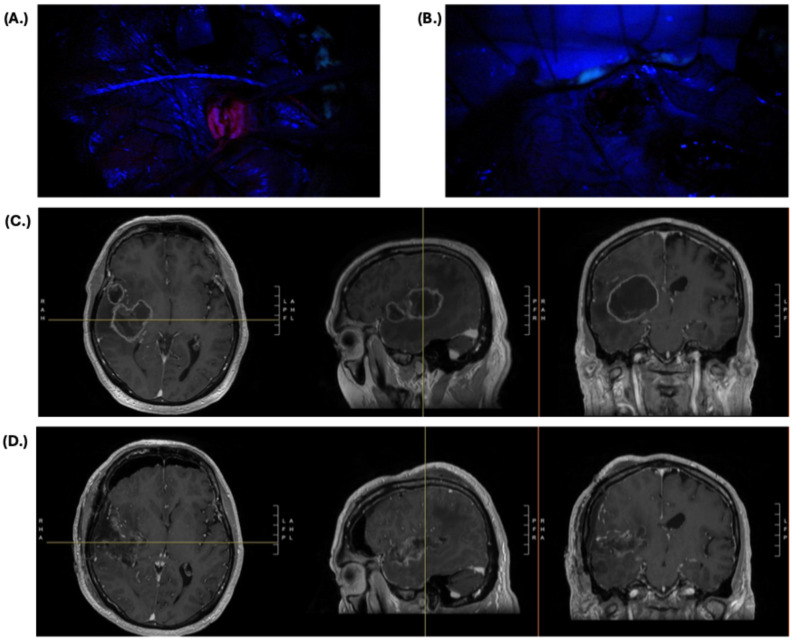
Case example of a 71-year-old patient with a 5.2 cm × 5.0 cm × 4.2 cm (AP × TV × CC) right-sided, peripherally enhancing, cystic, temporo-insular mass suspicious for high-grade glioma and associated with vasogenic edema, 1.0 cm midline shift, and left-sided weakness. The patient underwent a right-sided craniotomy with 5-ALA fluorescence guidance and motor mapping with final pathology consistent with IDH-wildtype, WHO grade IV glioblastoma. In the setting of an IDH-wildtype glioblastoma, (**A**) 5-ALA allowed for bright, visible fluorescence intraoperatively prior to resection, and, (**B**) following completion of resection, there was elimination of the fluorescent signal in non-eloquent tissue as identified on motor mapping. Preoperative MRI T1 with contrast images is presented in panel (**C**), and, in the postoperative MRI in panel (**D**), the resection of all tissue that could be safely resected is visualized, which was facilitated by the use of both 5-ALA and motor mapping.

The use of 5-ALA in neurosurgical oncology has made significant strides over the past two decades, with many advances already being made to enhance the sensitivity, visibility, and interpretation of fluorescence across different tumors. However, there is still room to improve in the realm of increasing maximal 5-ALA fluorescence to avoid false-negative signal. In some studies, this rate is as high as 60–70% for non-fluorescent samples and is especially concerning at the tumor’s margins where the tumor cell density and fluorescence signal are intrinsically lower and more heterogenous [52,89]. As a result, experimental therapies are being developed, and other technologies are being tested in combination with 5-ALA to increase tumor detection. One strategy has been the modulation of heme biosynthesis and transit to artificially increase the maximal accumulation of PpIX. Downregulation of ferrochelatase at the gene expression level through siRNAs as well as the use of iron chelators to reduce intracellular iron levels have resulted in increased PpIX accumulation and fluorescence in vitro [90,91]. Similarly, upregulation of both ABCB6, the transport protein for the precursor coproporphyrinogen III (CPgenIII), and pretreatment with calcitriol, a modulator of the enzyme that synthesizes CPgenIII, also increased PpIX levels and signal intensity [92,93]. However, all of these mechanisms have yet to be tested clinically, so their true efficacy remains unclear. What has been more tangible for increasing tumor detection is the use of intraoperative tools that quantify the degree of fluorescence or PpIX concentration. Intraoperative spectroscopic handheld probes that translate collected light wavelengths into tissue PpIX concentration measurements have consistently increased the detection rate of previously unnoticed tumor regions [62,63,72]. Finally, perhaps one of the most promising developments has been the combinatorial use of 5-ALA with other validated modalities intended to achieve maximal safe resection. There have been many studies evaluating the use of 5-ALA with functional brain mapping, which has allowed for more complete resections in eloquent brain areas without exacerbating morbidity [94,95,96,97]. The increased availability of intraoperative MRI has also permitted studies into its dual use with 5-ALA, which have demonstrated increased rates of gross total resection [98,99,100]. The question of whether new technologies known to disrupt the blood–brain barrier such as focused ultrasound and laser interstitial thermal therapy can increase transit of 5-ALA to the tumor given its known mechanism of action has yet to be explored. Further investigation into whether the dual use of these tools increases survival and how to standardize their use and patient selection so that it is cost effective still needs to be performed; however, the preliminary data and experience are promising for the treatment of future brain tumor patients. At our own institution, 5-ALA is routinely applied in conjunction with other modalities, most commonly functional brain mapping, and results in good outcomes. An example of a patient treated by the authors with left hemibody weakness due to a right temporo-insular IDH-wildtype glioblastoma is depicted in Figure 1. This patient underwent an asleep craniotomy with 5-ALA and cortical and subcortical motor mapping. At discharge, the patient had regained full strength in his left hemibody and had near total resection of all the tumor that could be safely resected. Both 5-ALA and motor mapping were critical to this patient’s outcome. 5-ALA demonstrated the entire tumor burden in real time while the motor mapping helped determine whether the visualized tumor was safe for resection given its intimate association with the eloquent motor tracts. This case emphasizes the importance of combining 5-ALA and functional mapping, where the former allows for maximal resection and the latter mitigates morbidity.

One of the other main challenges in the use of 5-ALA is making the distinction between true-positive and false-positive signals. While 5-ALA is used in tumor recurrence, the issue of false-positive signals is a major issue in these settings as 5-ALA fluorescence has been demonstrated even in the absence of tumor cells and at the single-cell level in non-neoplastic tissue [101]. This primarily occurs because of gliotic tissue, reactive astrocytes, immune cells, and necrotic scar and cellular debris, all of which largely develop secondary to surgery and adjuvant treatments including radiation and chemotherapy [89,102,103]. The mechanism by which 5-ALA accumulates in these non-oncologic tissue remains unclear, but the working hypothesis is that reactive glial cells and astrocytes cause blood–brain barrier dysfunction, allowing for extracellular accumulation of 5-ALA. Immune cells including macrophages, histiocytes, and lymphocytes then all have mechanisms of internalizing and metabolizing 5-ALA similar to tumor cells, such as higher levels of enzymatic precursors in the PpIX pathway [102,104,105,106]. While this continues to be a major issue in the use of 5-ALA, there are some promising findings that could lead to promising solutions. First, multiple studies have shown that the wavelength spectra of fluorescence emitted from non-tumor tissue differs from the wavelength emitted from cancer cells. These changes can be detected by wavelength spectroscopy or multiphoton microscopy, which could be incorporated into the workflow of 5-ALA use in recurrent gliomas [106,107]. Another possibility is the pharmacologic modulation of 5-ALA leakage into the extracellular space, where inhibition of ABCG2 by lapatinib decreases efflux from tumor cells and decreases the heterogeneity of 5-ALA fluorescence by consolidating accumulation in tumor tissue [108]. The most promising solution, however, is the incorporation of the up-and-coming stimulated Raman histology technology with 5-ALA. The most definitive assessment of true- versus false-positive tissue is histopathological confirmation, which traditionally is performed by a neuropathologist but has significant logistical obstacles due to requiring tissue transportation, sectioning, staining, and reading by the pathologist, who may not always be available. The recent advent of stimulated Raman histology in combination with artificial intelligence technology has bypassed these challenges and allowed for accurate intraoperative diagnoses of suspected pathologic tissue [109]. Already, the combinatorial use of 5-ALA and Raman spectroscopy has increased tumor detection accuracy relative to both modalities in isolation, and, just recently in 2024, Nasir-Moin et al. demonstrated that a microscope with stimulated Raman histology and two-photon excitation fluorescence microscopy paired together improved the discrimination of florescent signal within tissue and between different cell types [110,111].

Finally, while 5-ALA has been extensively tested in the treatment of brain metastases, its specific role remains undefined given its extremely variable fluorescence across different primary cancer types. The rate of visible fluorescence ranges between 60 and 70% in the largest patient cohorts [112,113,114,115]. While it is well known that the highest rate of fluorescence in brain metastases is in tumors of epithelial origin, including breast, colon, lung, and melanoma, there is still extensive heterogeneity amongst those groups, with rates of fluorescence ranging from 50 to 80% [113,116]. Overall, a better understanding and ability to predict which metastatic brain tumors will fluoresce in the setting of 5-ALA are required before it can become routinely incorporated into the treatment armamentarium for brain metastases like it has become for HGGs and is trending towards for LGGs and meningiomas.

## 5. Conclusions

Over the past two decades, 5-ALA has become FDA approved and is routinely incorporated into the treatment of high-grade gliomas to maximize gross total resection. There is growing evidence to suggest that 5-ALA can assist in the surgical treatment of meningiomas by increasing extent of resection to include previously undetected disease as well as low-grade gliomas by identifying tumor foci that have transformed into malignant disease and have high risk of recurrence. The role of 5-ALA in brain metastases is unclear due to the significant heterogeneity in visible fluorescence across different primary cancers and within similar cancer groups. Additional research is needed to improve the efficacy of 5-ALA in neurosurgical oncology, particularly through clinical trials that compare different dosages and timing of 5-ALA administration and investigate new pharmacologic therapies and devices that increase the sensitivity and specificity of 5-ALA in all brain tumors. However, significant progress has already been made in optimizing intraoperative interpretation of 5-ALA fluorescence and combining its use with other technologies like functional brain mapping, intraoperative MRI, and stimulated Raman histology, all of which have increased its generalizability and ability to accomplish both maximal and safe surgical resection.

## Figures and Tables

**Table 1:** Properties, applications, and advantages of the most commonly used fluorophores in neurosurgery.

Fluorophore	Characteristics	Applications	Advantages
5-ALA	Excitation: 375–440 nm Emission: 630–720 nm Administration: Oral Half-Life: 1–3 h Latency Period: 7–9 h	- High-grade gliomas; - Low-grade gliomas; - Meningiomas; - Brain metastases.	- Most reliable fluorescence in high-grade gliomas; - Most reliable fluorescence in low-grade gliomas, albeit still heterogenous; - Longest duration (hours) of fluorescence; - FDA approved for high-grade gliomas; - Possible cytotoxic effects through photodynamic therapy.
Fluorescein	Excitation: 460–500 nm Emission: 540–690 nm Administration: IV Half-Life: 23.5 min Latency Period: 2–4 h	- Brain metastases; - High-grade gliomas; - Meningiomas.	- Most reliable fluorescence in brain metastases; - Most reliable fluorescence of dural invasion in meningiomas; - Long duration (hours) of fluorescence; - Least expensive administration.
ICG	Excitation: 750–800 nm Emission: 700–850 nm Administration: IV Half-Life: 3–4 min Latency Period: Seconds	- AVMs; - Aneurysm; - Cavernous hemangioma; - Hemangioblastoma; - High-grade gliomas; - Meningioma; - Brain metastases.	- Visualizes tumor vasculature; - Near-infrared fluorescence, which allows for deeper penetrance and less autofluorescence; - Immediate fluorescence after administration, albeit short-lived.

5-ALA, 5-Aminolevulilnic Acid; AVM, Arteriovenous malformations; ICG, indocyanine green.

**Table 2:** Technical considerations in the use of 5-ALA for the most common brain tumor indications.

	High-Grade Glioma	Low-Grade Glioma	Meningioma
Rates of 5-ALA Fluorescence	83–96% [56,86,87]	22–56% [58,86,87,88]	77–94% [56,86,87]
Patient Factors	Preoperatively evaluate for - History of acute or chronic porphyria (do not use 5-ALA if positive); - History of liver dysfunction, cirrhosis, or adiposity (consider not using 5-ALA if positive); - Current use of anti-hypertensive, hepatotoxic, or phototoxic medications (consider holding at least 24 h prior to surgery); - Baseline liver function tests; - Probability of high-grade versus low-grade glioma.		
5-ALA Dose	20 mg/kg	At least 20 mg/kg, but consider up to 40 mg/kg for maximal fluorescent intensity	20 mg/kg
Timing *	3–5 h prior to anesthesia induction	3–5 h prior to anesthesia induction	No more than 3–5 h prior to anesthesia induction and as low as 1–3 h prior can be considered depending on anticipated time to dural incision
Tumor-Specific Intraoperative Adjuncts Ψ	Primary or recurrent: - Adapted instruments for deep-seated lesions; - Functional brain mapping for eloquent lesions; Recurrent tumor: - Raman spectroscopy for distinguishing between tumor, pseudoprogression, and radiation necrosis.	Primary or recurrent: - Handheld spectroscopic probe for maximizing detection of fluorescence; - Adapted instruments for deep-seated lesions; - Functional brain mapping for eloquent lesions.	Raman spectroscopy for distinguishing between pathologic dural tail and dura versus healthy dura
Postoperative Precautions	- Limit patient light exposure for 48 h postoperatively; - Repeat liver function tests to evaluate for liver injury; - Supportive management for possible hypotension, nausea, vomiting, and diarrhea.		

* Most important consideration is neurosurgeon’s anticipated time to tumor debulking. Ψ Conditional on institution availability.
